# Response of soil organic carbon fractions, microbial community composition and carbon mineralization to high-input fertilizer practices under an intensive agricultural system

**DOI:** 10.1371/journal.pone.0195144

**Published:** 2018-04-18

**Authors:** Jing Li, Xueping Wu, Mesfin Tsegaye Gebremikael, Huijun Wu, Dianxiong Cai, Bisheng Wang, Baoguo Li, Jiancheng Zhang, Yongshan Li, Jilong Xi

**Affiliations:** 1 Ministry of Agriculture Key Laboratory of Crop Nutrition and Fertilization, Institute of Agricultural Resources and Regional Planning, Chinese Academy of Agricultural Sciences, Beijing, P.R.China; 2 College of Resources and Environmental Sciences, China Agricultural University, Beijing, China; 3 Department of Soil Management, Ghent University, Coupure Links, Gent, Belgium; 4 Wheat Research Institute, Shanxi Academy of Agricultural Sciences, Linfen, P.R.China; 5 Cotton Research Institute, Shanxi Academy of Agricultural Sciences, Yuncheng, P.R.China; Beijing Normal University, CHINA

## Abstract

Microbial mechanisms associated with soil organic carbon (SOC) decomposition are poorly understood. We aim to determine the effects of inorganic and organic fertilizers on soil labile carbon (C) pools, microbial community structure and C mineralization rate under an intensive wheat-maize double cropping system in Northern China. Soil samples in 0–10 cm layer were collected from a nine-year field trial involved four treatments: no fertilizer, CK; nitrogen (N) and phosphorus (P) fertilizers, NP; maize straw combined with NP fertilizers, NPS; and manure plus straw and NP fertilizers, NPSM. Soil samples were analyzed to determine labile C pools (including dissolved organic C, DOC; light free organic C, LFOC; and microbial biomass C, MBC), microbial community composition (using phospholipid fatty acid (PLFA) profiles) and SOC mineralization rate (from a 124-day incubation experiment). This study demonstrated that the application of chemical fertilizers (NP) alone did not alter labile C fractions, soil microbial communities and SOC mineralization rate from those observed in the CK treatment. Whereas the use of straw in conjunction with chemical fertilizers (NPS) became an additional labile substrate supply that decreased C limitation, stimulated growth of all PLFA-related microbial communities, and resulted in 53% higher cumulative mineralization of C compared to that of CK. The SOC and its labile fractions explained 78.7% of the variance of microbial community structure. Further addition of manure on the top of straw in the NPSM treatment did not significantly increase microbial community abundances, but it did alter microbial community structure by increasing G+/G- ratio compared to that of NPS. The cumulative mineralization of C was 85% higher under NPSM fertilization compared to that of CK. Particularly, the NPSM treatment increased the mineralization rate of the resistant pool. This has to be carefully taken into account when setting realistic and effective goals for long-term soil C stabilization.

## Introduction

Soil organic carbon (SOC) is recognized as the largest terrestrial carbon (C) reservoir and has gained much attention because of its importance to soil fertility, crop productivity, and climate change mitigation [1, 2]. Fertilization is an important determinant of quantities of SOC in croplands since it can change the equilibrium between primary C inputs and C decomposition [3, 4]. Soil microorganisms are the main decomposers of SOC and key drivers of soil nutrient cycling in agricultural eco-systems [5]. A better understanding of the mechanisms of SOC decomposition via microorganisms is critical for identifying fertilization strategies that maintain and improve soil C accumulation and soil fertility.

The changes of soil microbial communities under different fertilization regimes may be contributed to changes in environmental characteristics, such as soil water content [6], temperature [7], pH [8] and substrate availability [9]. The content and quality of SOC are considered key factors that affect soil microbial communities [9]. However, increases in SOC content following the addition of fertilizers may take considerable time. Consequently, changes in SOC cannot fully and quickly reflect the influence that the complexity of the organic compounds may have on the microbiological processes controlling nutrient availability. Soil labile C fractions are a series of small, but sensitive, proportions of SOC with turnover times of a few days to months. It was revealed that soil labile C fractions, like dissolved organic C (DOC) and microbial biomass C (MBC) were major determinants for the preservation of soil microbial diversity in long-term fertilization trials [9]. Most studies documented that the application of organic manure increased the amounts of labile organic C pools [10, 11]. However, no influences results have also been reported [12]. The inconsistent results may be attributed to the differences of input sources and rate of fertilizers, tillage management, crop rotation, experiment duration, and site-specifics [12–14].

The effects of chemical fertilizers and organic amendments on soil microorganisms have been given particular attention. A meta-analysis of long-term inorganic fertilizer trials revealed a 15.1% increase of the microbial biomass after mineral fertilizers application compared to unfertilized treatments [15]. Eo and Park [16] also found that inputs of nitrogen (N) and phosphorus (P) fertilizers had considerable effects on specific bacterial groups. Furthermore, it is generally accepted that organic fertilizers have more significant effect on abundances of microorganisms in soils compared with mineral fertilizers [17, 9]. Ngosong et al. [18] observed that organic manure increased fungal abundance especially that of arbuscular mycorrhizal fungi (AMF). Moreover, Elfstrand et al. [19] found higher fungi /bacteria ratios (F/B) in soils receiving green manure. Changes in microbial community structures in turn have important implications for the SOC mineralization. For example, Lipson et al. [20] stated that bacteria had higher growth rates and lower yields than fungi, suggesting a more important role for bacteria in determining soil heterotrophic respiration. However, Dai et al. [21] reported that alterations in soil microbial abundance and community composition did not significantly influence the C mineralization under long-term fertilization in paddy soils. The relationships between soil microbial community composition and function are not always straightforward because of the existence of several microbial groups that carry out similar functions and the complexity of soil system [22]. Thus, uncertainties still remain about the impacts of mineral and organic fertilization on soil microbial communities and their roles in SOC mineralization.

The winter wheat-summer maize double cropping is the principal cropping system in northern China, covering an area of 16 million hectares, where its outputs account for about a quarter of the total national food production [23]. In recent years, the productivity of soils in this cropping system has been declining as a result of unsustainable agricultural practices, such as frequent tillage, crop residue removal, and excessive mineral fertilizer application. Simultaneously, application of organic amendment to soil in the form of manure and straw is commonly recommended to improve SOC quality and quantity and increase crop yield. To be mentioned, annual organic C and mineral N inputs under the maize-wheat double cropping system can be multifold higher than single cereal-based rotations. Previous studies have shown that different fertilization regimes had profound impacts on crop yield and SOC contents under this cropping system [24, 25]. However, it is not clear how high-input fertilizer practices affect soil microorganisms and functions relevant to SOC decomposition. We hypothesized that (1) different fertilization methods affect microbial community structure because C and N inputs will directly and indirectly decrease possible nutrient limitation; and (2) these changes in microbial community composition will alter the SOC decomposition rate. Thus, the objectives of this study were to: (1) verify the effects of different fertilization methods on SOC labile fractions and abundance and composition of microbial communities; (2) determine the influences of different fertilization methods on SOC mineralization; and (3) evaluate the roles of labile C fractions and soil microbial communities in SOC mineralization.

## Methods and materials

### Ethics statement

The study was conducted at the Niujiawa Agricultural Experimental Farm of Shanxi Academy of Agricultural Sciences located in Yuncheng city, Shanxi province, China. Permission was obtained from the administration to allow soil sampling. No rare or endangered wild animals were collected in this experiment. Furthermore, this study did not use wild animals as research objects and did not threaten the environmental system.

### Study area and experiment design

A field experiment with a winter wheat (*Triticum aestivum L*.) and summer maize (*Zea mays L*.) double-cropping system had been conducted since 2007 at the Niujiawa Agricultural Experimental Farm located in Yuncheng city, Shanxi province, China (35°11′ N, 111°05′ E). The climate is temperate monsoonal with an average annual temperature of 13.3°C and an average annual rainfall of 525 mm. The soil with a silty clay loam texture (17.5% clay, 28.0% sand and 54.5% silt) in the upper surface horizon was developed from alluvial sediments of the Yellow River. Its mean pH is 8.15, CaCO_3_ content is 6.5%, and bulk density is 1.39 g cm^-3^ in the 0 to 10 cm soil layer.

The field experiment was conducted based on a completely randomized design with three replications of each treatment. The size of each plot was 60 m^2^. The four treatments were (1) CK, unfertilized control; (2) NP, inorganic N and P fertilizers; (3) NPS, mineral N and P fertilizers in combination with maize straw; and (4) NPSM, mineral N and P fertilizers in combination with both maize straw and chicken manure. For the NP, NPS and NPSM treatments, mineral N and P fertilizers were applied in the forms of urea and calcium super-phosphate, which totally supplied 450 kg N ha^-1^ yr^-1^and 148.5 kg P ha^-1^ yr^-1^ to two crops. Two-thirds of urea and total amounts of calcium super-phosphate and manure were applied as a basal dose before sowing, the remaining one-third of urea was used at the jointing stage of each crop. For the NPSM treatment, 9 t ha^-1^ yr^-1^ (dry weight) of chicken manure was added to each crop. The organic C and total N contents of the manure were 233 and 19.9 g kg^-1^, respectively, those totally supplied roughly 4.2 t C ha^-1^ yr^-1^ and 358 kg N ha^-1^ yr^-1^ to two crops. For the NPS and the NPSM treatments, 10.5 t ha^-1^ yr^-1^ (dry weight) of maize straw chopped into 10 cm length pieces, was returned to the soil prior to wheat sowing. The C and N contents of maize straw were 336 and 6.3 g kg^-1^, which approximately supplied 3.5 t C ha^-1^ yr^-1^ and 66 kg N ha^-1^ yr^-1^. Standing crop above ground parts were harvested at soil surface and removed from the CK and NP plots. The wheat straw of all treatments was incorporated into soils after being chopped. A rotary tillage operation till was performed by machine before sowing and for mixing of mineral fertilizers, straw and manure into soil at about a 10–15 cm depth.

### Soil chemo-physical analysis

In March 2016, composite soil samples (500g) from each plot were collected from the 0–10cm soil layer at ten randomly chosen points. Finely ground soil samples were treated with 1.0 M HCl for 24 h at 20°C to remove inorganic C before analysis [26]. The contents of SOC and total N were measured using an elemental analyzer (Vario EL II, Germany). The DOC and dissolved organic N (DON) was extracted with distilled water (1:5 soil: water) by the method described by Gong et al. [24]. After removing inorganic C by using concentrated phosphoric acid, the amount of organic C in the filtrate was determined by a TOC analyzer (Vario TOC, Germany). The DON was calculated as the difference between total dissolved N and inorganic N in the filtrate [27]. Light free organic C (LFOC) and light free N (LFN) was determined by adding sodium polytungstate solution with a density of 1.6 g cm^-3^ according to the method of Dorodnikov et al. [28]. Organic C and N contents of light free fractions were measured using an elemental analyzer (Vario EL II, Germany). The MBC and microbial biomass N (MBN) were determined by the chloroform-fumigation extraction method based on the difference between chloroform-treated and untreated soils [29]. The organic C and N concentrations of K_2_SO_4_-extracted solutions were measured using a TOC analyzer (Vario TOC, Germany).

### Microbial community structure

Soil microbial community composition was determined by phospholipid fatty acid (PLFA) analysis as described by Ai et al. [30]. Three grams of freeze-dried soil samples were used to extract the lipids by a single-phase CHCl_3_: methanol: citrate buffer (15.2 mL at a 1:2:0.8 volume ratio). The soil extracts were fractionated into neutral lipids, glycolipids, and polar lipids using a silica-bonded phase column (SPE-Si, Supelco, Poole, UK) with CHCl_3_, acetone and methanol, respectively. The recovered polar lipids were saponified and methylated to fatty acid methyl esters (FAME). FAMES were quantified by a gas chromatograph (N6890, Agilent) and identified with an MIDI Sherlock Microbial Identification System (Version 4.5, MIDI, Inc., Newark, DE).

We divided all PLFAs into 5 microbial groups based on previously published PLFA biomarker data [31]: Gram-positive (G+) bacteria (i14:0, i15:0, a15:0, i16:0, i17:0,a17:0), Gram-negative (G-) bacteria (16:1ω7c, 16:1ω9c, 17:1ω8c, 18:1ω5c, 18:1ω7c, cy17:0, cy19:0), actinomycetes (10Me16:0, 10Me17:0, 10Me18:0), saprophytic fungi (18:1ω9c and 18:2ω6c) and AMF (16:1ω5c). Bacterial sums were calculated using G+, G-, and actinomycete markers; fungal sums were calculated using both saprophytic and AMF fungal markers. Bacterial sums and fungal sums were used to calculate the ratio of fungal/bacterial PLFAs (F/B). (http://dx.doi.org/10.17504/protocols.io.nggdbtw)

### C mineralization

An incubation experiment was conducted at 25°C and 60% water-holding capacity for 124 days. In brief, each fresh soil sample of 25 g was put into a 250 ml glass jar. After one week of a pre-incubation period, CO_2_ samples were taken after 1, 3, 6, 9, 13 days, and then every 7 days interval up to 27 days, 14 days up to 55 days and later 24 days up to 124 days of incubation. Before taking CO_2_ samples, we closed the jars with air-tight seals for 24 h. The CO_2_ concentration was measured using a gas chromatograph (Agient 7890A). (http://dx.doi.org/10.17504/protocols.io.nhedb3e)

The cumulative amount of mineralized C (C_t_ in mg CO_2_-C kg^-1^ soil) produced in each treatment was calculated and was then plotted against incubation time (t). Furthermore, the C_t_ was fitted to a parallel first-plus-zero order kinetic model [32]:

$$C_{t}=C_{0}(1-e^{-k_{f}\times t})+k_{s}$$

This model assumes the existence of two pools of available C with different resistance against microbial degradation: (1) an easily mineralizable pool (C_0_) that mineralizes according to first order kinetics at a rate of *k*_*f*_ (mg CO_2_-C kg^-1^ day^-1^), and (2) a more resistant C pool that mineralizes according to zero order kinetics at a rate of *k*_*s*_ (mg CO_2_-C kg^-1^ day^-1^). All curves were fitted with SPSS software (version 23.0, Inc., Chicago, IL) as unconstrained non-linear regressions by the Levenberg–Marquardt algorithm.

### Statistical analysis

One-way ANOVA was used to test the effect of fertilizer treatments on the organic C content and C/N ratios of labile fractions, abundance of PLFA biomarkers, cumulative amount of emitted CO_2_-C, C_0_, *k*_*f*_ and *k*_*s*_. Differences between individual means were tested by Tukey HSD post hoc tests. The magnitudes of correlations among soil C characteristics, microbial communities and C mineralization parameters, were tested by Pearson correlation coefficient. Both statistical analyses above were carried out by SPSS software (version 23.0, Inc., Chicago, IL). Redundancy analysis (RDA) was applied to visualize the effect of soil labile organic fractions on microbial community structure, and was carried out using CANOCO software (version 5.0, Microcomputer Power, Inc., Ithaca, NY). In all analyses, statistical significance was recognized at *P*<0.05.

## Results

### SOC and soil labile organic C fractions

**Fig 1:**
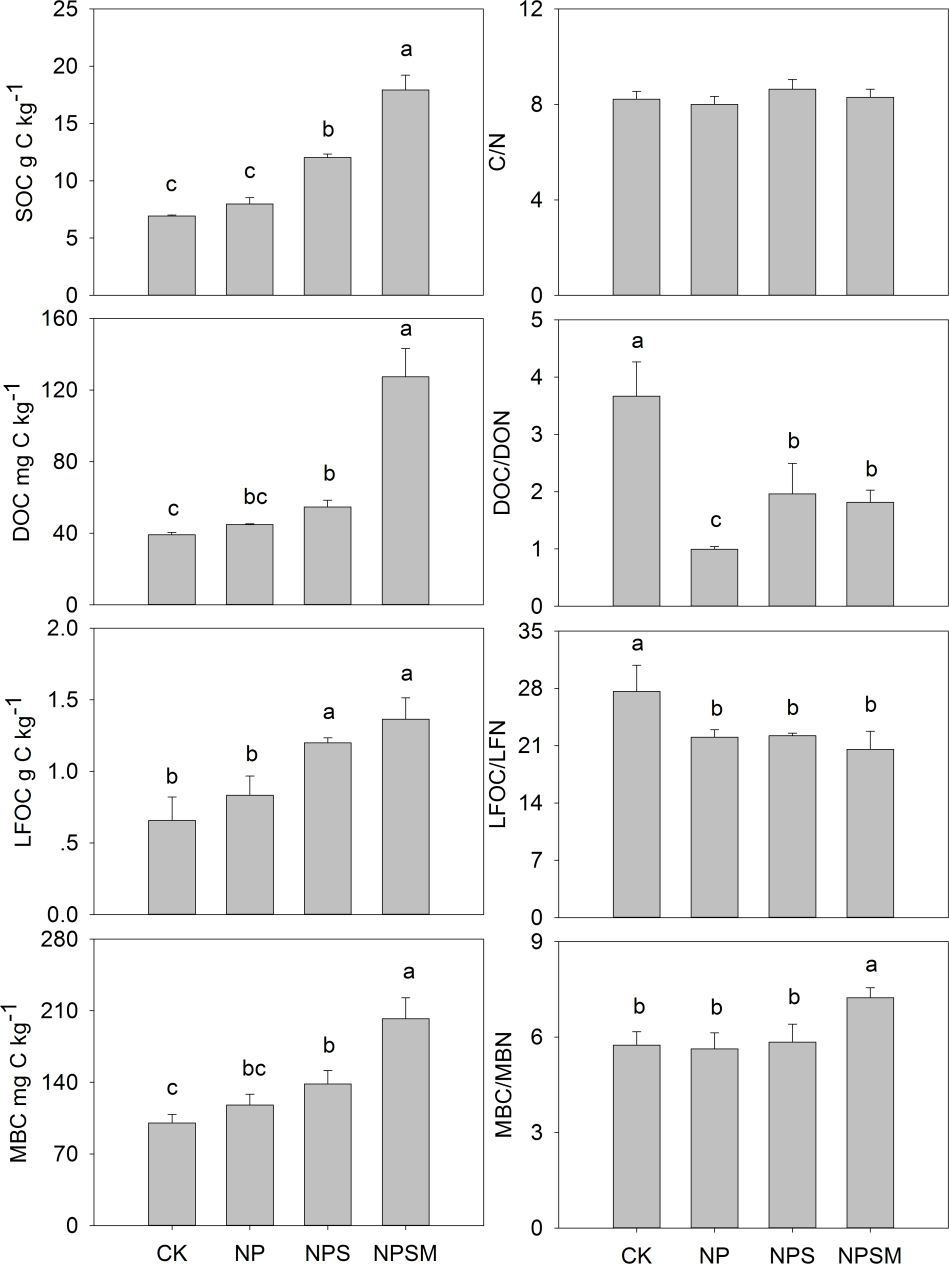
Organic C contents and C/N ratios of bulk soil and labile fractions under different fertilization regimes. Error bars represent standard error of the means (n = 3).

After nine years application of organic fertilizers, straw with manure (NPSM) or straw only (NPS) substantially increased SOC content by 143% and 71% (*P*<0.05), respectively, while application of chemical fertilizers alone did not affect SOC level compared with that of CK (Fig 1). The effects of fertilization on soil labile organic C showed a similar trend to total SOC. The contents of DOC, LFOC, and MBC were respectively 264%, 108%, and 102% higher after NPSM application, and respectively 57%, 82% and 38% higher after NPS application than compared with those of CK.

The C/N ratio of bulk soil was constant across all fertilization treatments, but C/N ratio of labile organic C factions had differential responses to the different treatments (Fig 1).Ratios of DOC/DON and LFOC/LFN were lower in treatments with additions of exogenous organic amendment and chemical fertilizers than in the control. In contrast, the MBC/MBN was 19% higher under NPSM application than compared to that of CK.

### Microbial community composition

**Fig 2:**
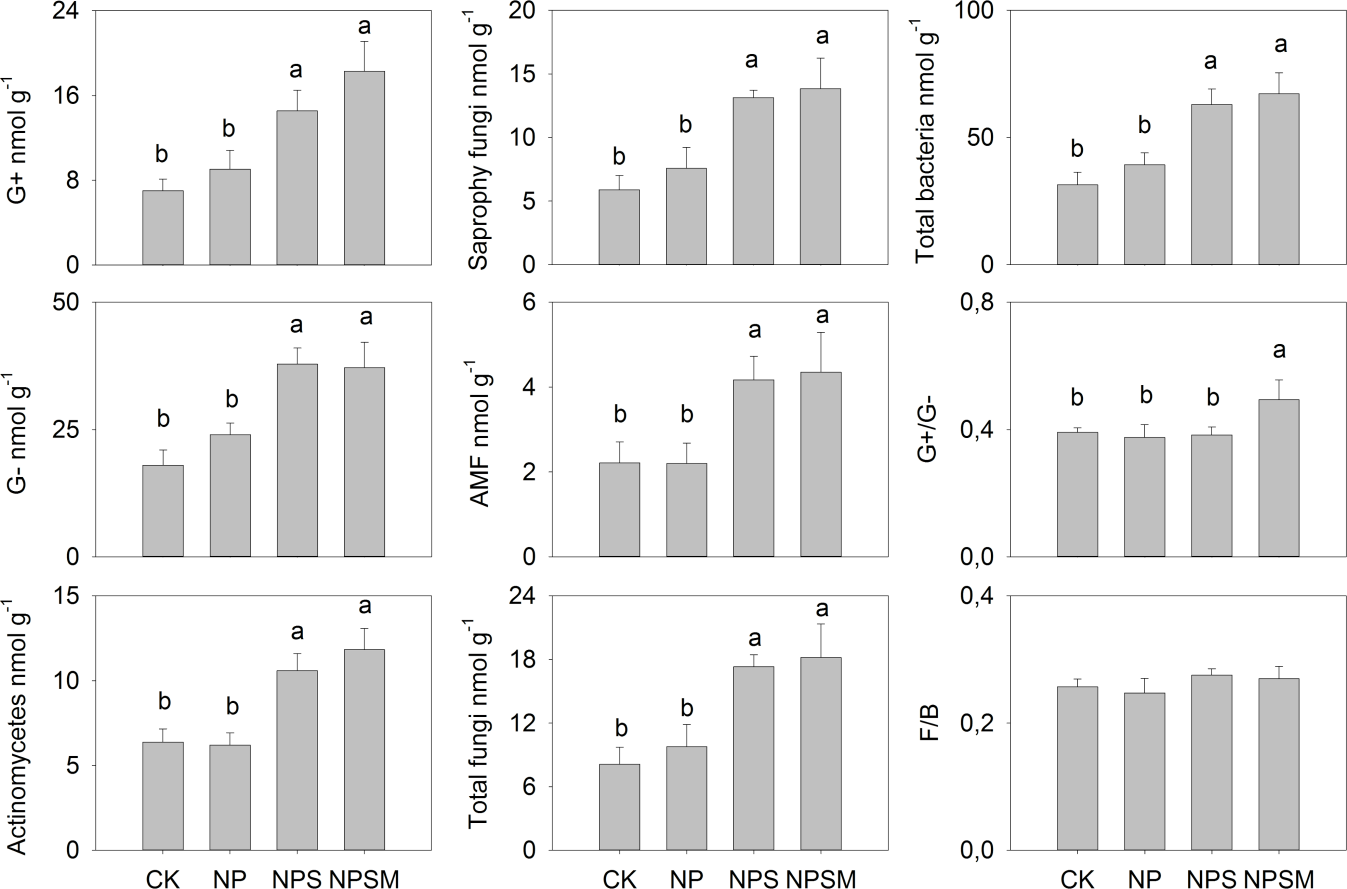
Abundance of microbial biomarker groups under different fertilization regimes. Error bars represent standard error of the means (n = 3).

Overall, the application of organic fertilizers significantly increased total PLFAs abundance. We calculated abundances of specific microbial groups for data analysis to determine the microbial community composition under various fertilizer treatments. The NPSM and NPS fertilization treatments had significantly greater abundances of all microbial groups considered (i.e. G+, G-, actinomycetes, saprophytic fungi and AMF), however, we found no further increases from NPS to NPSM (Fig 2). Compared with CK, NPSM and NPS treatments caused greater measures of G+ and G- biomarkers by 107–160% and 106–110%, and greater measures of actinomycetes by 66–86%. The NPSM and NPS treatments were also greater in abundances of fungal communities, the saprophytic fungi were greater by 123–135% and AMF was greater by 88–96%. The G+/G- ratio was higher under NPSM treatment compared to other treatments, indicating that NPSM fertilization had changed soil microbial communities. However, there were no obvious differences of F/B ratios across all treatments.

**Fig 3:**
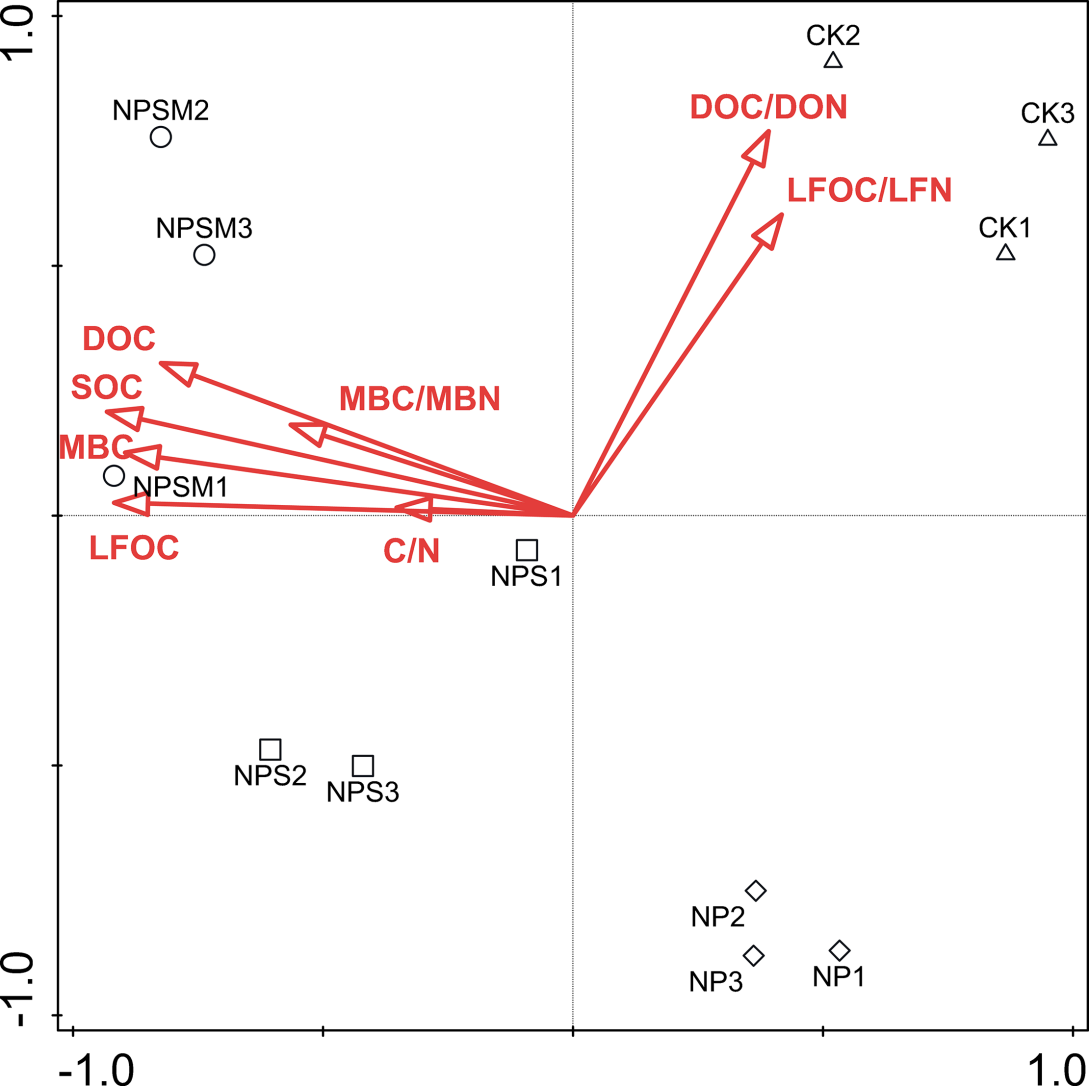
Redundancy analysis (RDA) of the soil microbial communities constrained by labile organic C fractions.

Correlations between microbial community structure and labile organic C fractions were analyzed by the RDA plot (Fig 3). Soil labile fractions were used as environmental variables. The first and second ordination axes accounted for 75.33% and 3.38%) of the total variation between soil C fractions and microbial community composition assessed by PLFAs. The C contents of bulk soil and all labile fractions were significantly (*P*<0.05) and positively correlated with microbial communities along the first axis, while DOC/DON and LFOC/LFN showed negative correlation with microbial communities.

### Soil C mineralization

**Fig 4:**
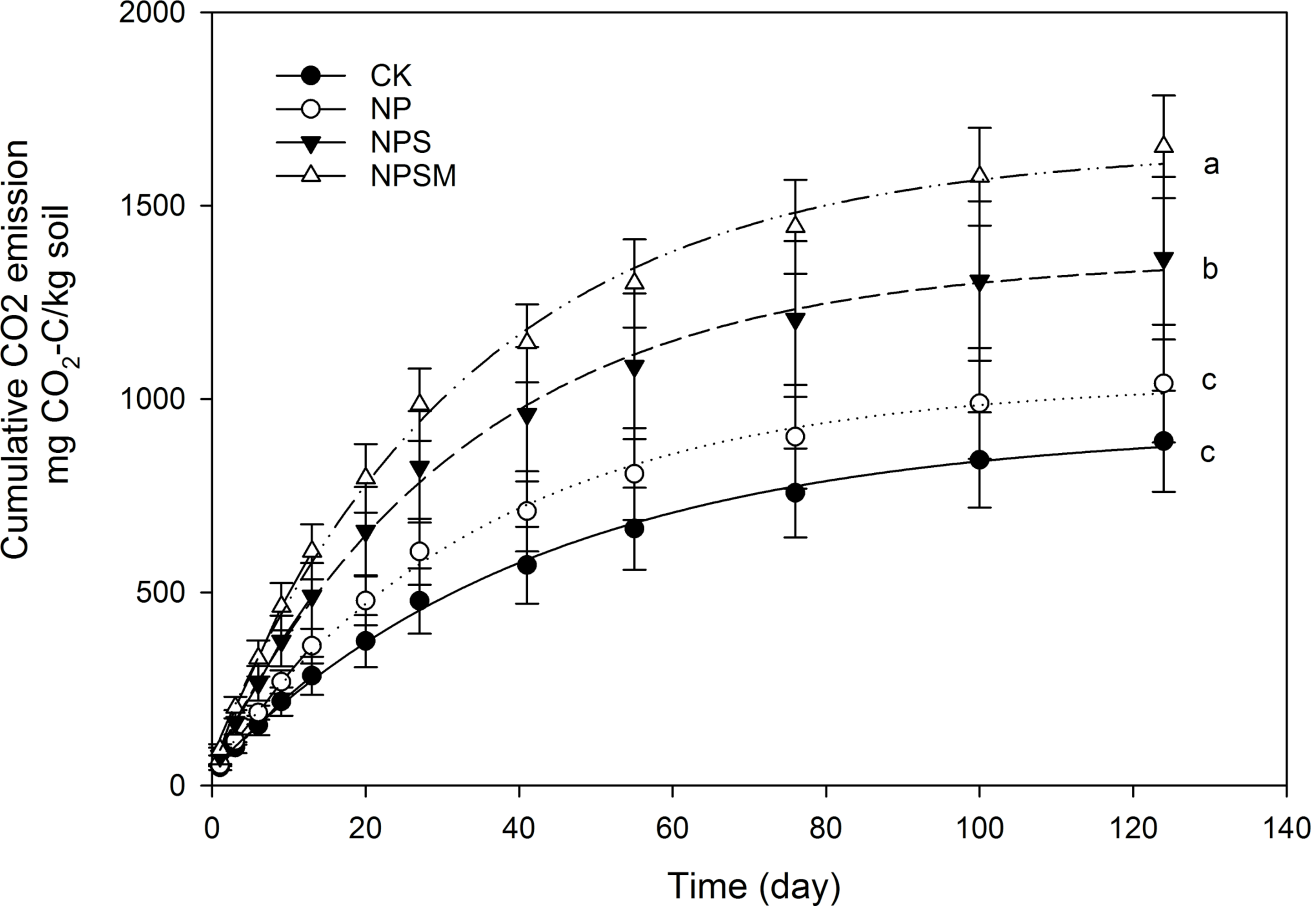
Cumulative CO_2_ emission over time under different fertilization regimes. Error bars represent standard error of the means (n = 3).

Cumulative CO_2_-C emission over time tended to be higher in NPSM and NPS than in CK and NP treatments throughout the incubation period (Fig 4). By the end of the incubation, the NPSM treatment had largest increase of the cumulative mineralization C (C_min_) by 85%, and the NPS treatment also resulted in an increase of 53% (Table 1) compared to CK. (Table 1).

**Table 1:** The parameters of curve fittings for all mineralization data using a parallel first-plus-zero-order kinetic model. Values are the means±SD (n = 3).

Treatment	C_min_ (mg CO_2_-C kg^-1^)	C_0_ (mg CO_2_-C kg^-1^)	*k*_*f*_ (day^-1^)	*k*_*s*_ (mg CO_2_-C kg^-1^ day^-1^)	R^2^
CK	891±131c	553±104b	0.046±0.001a	2.82±0.29b	0.998
NP	1040±152c	717±136b	0.047±0.004a	2.68±0.38b	0.999
NPS	1364±149b	987±147a	0.048±0.001a	3.33±0.31b	0.998
NPSM	1653±133a	1122±81a	0.052±0.007a	4.42±0.6a	0.999

Values followed by same letters within each column are not significantly different (*P*<0.05)

Table 1 showed the results of curve fittings for all mineralization data using the first-plus-zero order kinetic model, where values of R^2^ that were close to 1, indicated that the model described the mineralization process satisfactorily. Compared to CK, the NPSM and NPS treatments enlarged the size of the easily mineralizable C pool (C_0_) by 103 and 78%, respectively. Likewise, mineralization rates of the resistant C pool (*k*_*s*_) were higher in organic fertilization plots than in CK plots, though only significantly (*P*<0.05) in NPSM fertilization plots.

### Relationships between C mineralization, labile fractions and microbial community composition

The C_min_ and C_0_ were significantly and positively correlated with SOC and C contents of all labile fractions, but were not correlated with C/N ratios (Table 2). The C_min_ and C_0_ were also correlated with the abundances of G+, G-, actinomycetes, saprophytic fungi, AMF, G+/G-, and F/B. The *k*_*s*_ was positively correlated with the SOC, DOC, MBC, G+/G- and the abundance of most microbial groups except G-.

**Table 2:** Pearson correlation between soil C mineralization parameters, C contents and C/N of labile fractions and microbial communities (n = 12).

	C_min_	C_0_	*k*_*f*_	*k*_*s*_
SOC	0.895**	0.855**	0.488	0.667*
DOC	0.833**	0.734**	0.702*	0.708*
LFOC	0.798**	0.787**	0.419	0.537
MBC	0.859**	0.789**	0.678*	0.669*
C/N	0.211	0.240	0.157	0.164
DOC/DON	-0.321	-0.380	0.262	0.109
LFOC/LFN	-0.246	-0.371	-0.180	-0.211
MBC/MBN	0.559	0.465	0.600*	0.607*
G+	0.987**	0.962**	0.473	0.701*
G-	0.890**	0.921**	0.389	0.533
Actinomycetes	0.951**	0.931**	0.404	0.721*
Saprophy fungi	0.938**	0.937**	0.443	0.649*
AMF	0.950**	0.934**	0.406	0.694*
G+/G-	0.718**	0.639**	0.305	0.739**
F/B	0.613*	0.605*	0.065	0.554

** Correlation is significant at the 0.01 level

* Correlation is significant at the 0.05 level.

## Discussion

### Effects of different fertilization regimes on SOC and labile organic C fractions

Nine years application of inorganic fertilizers alone did not significantly increase the bulk SOC level (Fig 1), indicating that additional C inputs derived from increased plant growth as a potential result of chemical fertilization were still offset by C loss from microbial decomposition. In line with previous reports [12], the return of straw (NPS) is an effective practice to increase SOC sequestration compared with chemical fertilization alone (NP) (Fig 1). In a meta-analysis of Wang et al. [33], the sequestration efficiency of straw-C ranged from -8.3 to 56.6% over 10–30 years, and on average, 9.5±1.1% of straw-C input was converted to SOC during a mean experimental period of 18 years in China. It is worth to note that the addition of manure on top of the straw in the NPSM treatment even further improved the amount of stored soil C (Fig 1), suggesting that tested soils have strong potentials to sequester considerable C from manure in a field with additional straw inputs.

Changes in labile organic C fractions can respond to soil management practices more quickly than total SOC content [24]. It has been widely accepted that application of organic manure markedly increases labile organic C fractions [24, 34, 35], which is consistent with our findings. The DOC is mobile within the soil solution and is thus considered to be the most bioavailable source of C substrates for microbial populations [36]. However, our results and that of Li et al. [37] suggested that application of chemical fertilizers alone had no significant effect on DOC, confirming that the primary source of DOC was organic amendments. Moreover, the NPSM treatment resulted in a sharp elevation of DOC compared to that of the NPS (Fig 1), which could be explained possibly by the presence of a considerable amount of soluble materials in manure amendments [38]. The LFOC represents the slightly decomposed plant litter [39] and functions as nucleation sites for fungi and other soil microbes [28]. In the current study, the straw or manure amendment directly contributed to the higher LFOC contents as light fraction dry matter (data not shown) and C contents were higher under NPS and NPSM treatments. The microbial biomass C is indicative of the size of the microbial biomass [37]. Significant increases of MBC were observed after manure or straw addition, suggesting that organic amendments had beneficial effects on growth of the microbial biomass probably by providing a readily-available source of C substrate and improving the soil environment [40].

The effects of fertilization on the C/N ratios of labile organic C pool have not been consistent, as indicated by the positive [41], negative [42] and lack of effects [36, 43] that have been reported. These inconsistent results may be attributed to the specific processes governing C and N cycling under specific fertilization practices. In the present study, the C/N ratio of total soil appeared to be stable, however, both chemical and organic fertilizers caused declines in DOC/DON and LFOC/LFN compared to those of the CK (Fig 1). The larger N input originating from urea, crop residues and manure resulted in decreases in C/N ratios of labile fractions. The MBC/MBN in NPSM treatment is comparable to the global average reported by Xu et al. [44]. The changes of MBC/MBN could be related to changes in microbial species and populations [42].

### Effects of different fertilization regimes on microbial community structure

Phospholipid fatty acids are major constituents of membranes of all living cells, and certain microorganisms groups have different “signature” fatty acids. Several studies have documented significant differences in the abundance and composition of soil microbial communities among the different fertilizer management programs based on PLFA patterns [30, 45]. In line with previous reports [30, 45], our results showed distinctly (*P*<0.05) higher amounts of total PLFAs and identified representative PLFA biomarkers in soils where organic fertilizers applied in combination with mineral fertilizers than compared to soils which received no organic fertilizers (Fig 2). Given that the microbial biomass is generally C limited in agricultural soil, the application of organic fertilizers presumably stimulated the growth of various microbial groups by increasing SOC labile fractions, which could act as major sources and energy for microorganisms [6]. This is further supported by the RDA data, which showed that C contents and C/N ratios of SOC and its labile fractions explained 78.7% of the variance of microbial community composition (Fig 3). Therefore, it is plausible that the addition of organic manure provided readily available substrates for the microbial community, whereas the relatively small increase of labile organic C under inorganic fertilization may be unable to support the substantial growth of microorganisms.

The microbial community composition at the experimental site was dominated by bacteria, which contributed 60% of total PLFAs. Lazcano et al. [46] described that bacteria were the most sensitive microbial groups to the different fertilizers because bacteria have a much shorter turnover time than fungi and can react faster to the environmental changes in soil. Our results showed that fertilization not only influenced the soil microbial abundances but also altered bacterial community composition. The G+ and G- bacteria have been reported to have different effects on C cycling and accumulation in soil, and the greater ratio of G+/G- is favorable for SOC accumulation [47]. Thus far, there have been inconsistent results concerning the effects of fertilization regimes on the abundance of G+ and G-. Peacock et al. [45] reported that a 5-year addition of manure increased the G- bacterial biomass but decreased the G+ bacterial biomass. In contrast, Ai et al [30] documented that a 31-year application of organic fertilizer increased the abundance of G+ bacteria, but application of chemical fertilizer increased G- bacteria [30]. In our study, a higher G+/G- ratio was found only in the NPSM fertilization treatment. Marschner et al. [48] also found higher G+/G- ratios under mineral fertilizer application with manure than with straw. Dong et al. [8] stated that microorganisms naturally occurring in applied organic matter amendments could also increase the overall greater microbial biomass. Thus, we assumed that the type of manure applied could be an important factor in determining G+/G-.

Fungi play an essential role in carbon and nutrient cycling in agricultural ecosystems and are known to be sensitive to fertilizers. In general, lower fungal biomass relative to bacteria is typical in agricultural eco-systems, and it has been attributed to different factors such as intensive physical disturbance, and altered amount of the nutrient inputs as compared to undisturbed soils [6, 49]. In the current experiment, low F/B ratios of PLFAs ranging from 0.25 to 0.28 for both mineral and organic fertilizers were observed. This is consistent with Ngosong et al. [18] who reported low values between 0.02 and 0.35 for mineral and organic (cattle manure) fertilizer amendments, respectively. Specifically, it has been reported that mineral fertilizers reduced F/B ratios, while organic manure stimulated fungi growth and thus increases F/B ratios [50]. However, we did not observe statistically significant differences in F/B ratios among our four different fertilization regimes. In support of our findings, Bardgett and McAlister [51] also found that applied fertilizers did not increase F/B ratios in a 6-year experiment, and they pointed out that this was due to high residual fertility.

### Effects of different fertilization regimes on C mineralization

As expected, the application of organic manure or straw combined with mineral fertilizers resulted in greater C mineralization than compared to application of mineral fertilizers alone or the unfertilized control. The result agrees with those by Mohanty et al. [52], who reported that long-term application of farmyard manure significantly and positively affected C mineralization in a rice-rice system. The mineralization of SOC is directly governed by interactions between the effects of microbial biomass, microbial community structure, substrate quality and availability, and microclimates [53, 54]. In our study, the soil samples from the treatments were incubated at the same temperature and moisture, which may have little effect on C mineralization. The relative importance in C mineralization was thus analyzed by the microbial communities and substrate availability (Table 2). We suggested that the C contents of SOC and its labile fractions, and microbial community abundance and composition were crucial for C mineralization, however, the C/N ratios of total soil and labile organic C fractions were less important for the C mineralization. These results were expected given that soil microbial community respiration is usually limited by the C substrates supply in the intensive agricultural systems.

Although all soils from the field sites were pre-incubated for one week, nevertheless, we still observed an initial flush of CO_2_ emission, most likely derived from rapid depletion of easily degradable organic C fractions (Fig 4). To better understanding of mineralization processes, we chose to model data by means of a parallel first-plus-zero-order kinetic model. In comparing the mineralization rate (*k*_*s*_) of the resistant pool among fertilizer practices, we found that *k*_*s*_ was 56.6% higher under the NPSM treatment than CK treatment. This has to be carefully taken into account when setting realistic and effective goals for long-term soil C stabilization. Positive and significant correlations were observed between *k*_*s*_ and the labile C fractions (DOC and MBC), indicating that differences in *k*_*s*_ values among fertilizer practices could be attributed to differences in the relative amounts of labile organic C fractions. We also found significant correlation between *k*_*s*_ and G+ bacteria, actinomycetes, saprophy fungi and AMF, but no significant correlation was found between *k*_*s*_ and G- bacterial. Marschner et al. [48] reported a shift in the response of G- and G+ bacteria to addition of exogenous organic material over time, indicating that G- bacteria were initially stimulated upon the addition of compost, while the abundance of G+ bacteria increased over time. Bastida et al. [55] observed that G- bacteria populations in a semiarid soil controlled the initial decomposition of soil organic matter, but fungi and, particularly actinomycetes played important roles in later steps following two months of incubation. However, our results were only speculated through the statistical approach. Future direct measurements are necessary to investigate how microbial communities and substrates are linked with C mineralization processes through dynamical studies.

## Conclusions

This study clearly indicated that nine years of various applied organic fertilizers significantly increased total SOC contents and labile organic C fractions (DOC, LFOC and MBC) in agricultural soils. Moreover, the greatest increases were observed in treatment with the combined applications of chicken manure, straw and mineral fertilizers. The application of organic fertilizers significantly increased abundances of all PLFA-related microbial communities including G+ bacteria, G- bacteria, actinomycetes, saprophytic fungi and AMF. Organic fertilization also slightly altered the composition of microbial communities. Furthermore, the application of organic fertilizers resulted in 53%-85% greater cumulative mineralization of C. Soil labile C fractions and soil microbial communities predominantly determined the variance in C mineralization, while C/N ratios of labile fractions did not significantly influenced C mineralization in the current agricultural system. This research provided information on the mineralization rate of resistant C pools, which are higher under organic fertilization regimes. This has to be carefully taken into account when setting realistic and effective goals for long-term soil C stabilization.
